# Estimating health-adjusted life expectancy conditional on risk factors: results for smoking and obesity

**DOI:** 10.1186/1478-7954-4-14

**Published:** 2006-11-03

**Authors:** Pieter HM van Baal, Rudolf T Hoogenveen, G Ardine de Wit, Hendriek C Boshuizen

**Affiliations:** 1 National Institute for Public Health and the Environment, Bilthoven, The Netherlands

## Abstract

**Background:**

Smoking and obesity are risk factors causing a large burden of disease. To help formulate and prioritize among smoking and obesity prevention activities, estimations of health-adjusted life expectancy (HALE) for cohorts that differ solely in their lifestyle (e.g. smoking vs. non smoking) can provide valuable information. Furthermore, in combination with estimates of life expectancy (LE), it can be tested whether prevention of obesity and smoking results in compression of morbidity.

**Methods:**

Using a dynamic population model that calculates the incidence of chronic disease conditional on epidemiological risk factors, we estimated LE and HALE at age 20 for a cohort of smokers with a normal weight (BMI < 25), a cohort of non-smoking obese people (BMI>30) and a cohort of 'healthy living' people (i.e. non smoking with a BMI < 25). Health state valuations for the different cohorts were calculated using the estimated disease prevalence rates in combination with data from the Dutch Burden of Disease study. Health state valuations are multiplied with life years to estimate HALE. Absolute compression of morbidity is defined as a reduction in unhealthy life expectancy (LE-HALE) and relative compression as a reduction in the proportion of life lived in good health (LE-HALE)/LE.

**Results:**

Estimates of HALE are highest for a 'healthy living' cohort (54.8 years for men and 55.4 years for women at age 20). Differences in HALE compared to 'healthy living' men at age 20 are 7.8 and 4.6 for respectively smoking and obese men. Differences in HALE compared to 'healthy living' women at age 20 are 6.0 and 4.5 for respectively smoking and obese women. Unhealthy life expectancy is about equal for all cohorts, meaning that successful prevention would not result in absolute compression of morbidity. Sensitivity analyses demonstrate that although estimates of LE and HALE are sensitive to changes in disease epidemiology, differences in LE and HALE between the different cohorts are fairly robust. In most cases, elimination of smoking or obesity does not result in absolute compression of morbidity but slightly increases the part of life lived in good health.

**Conclusion:**

Differences in HALE between smoking, obese and 'healthy living' cohorts are substantial and similar to differences in LE. However, our results do not indicate that substantial compression of morbidity is to be expected as a result of successful smoking or obesity prevention.

## Background

Obesity and smoking are risk factors for major chronic diseases that influence both length and quality of life [1]. Differences in life expectancy (LE) between smokers and never smokers have been found ranging from 7.5 years [2] to 10 years [3]. In a recent study, it was found that obesity led to decreases of roughly 6 to 7 years in life expectancy [4]. However, differences in LE alone are not sufficient to inform on the impact of unhealthy lifestyle since they do not address the impact on quality of life through disabilities caused by chronic diseases. To address this, summary measures of population health are needed that combine information on both length and quality of life [5]. One such summary measure of population health is health-adjusted life expectancy (HALE). HALE has been introduced within the Health Expectancy Network (Réseau Espérance de Vie en Santé, or REVES) and is a summary measure of population health indicating the expectation of equivalent years lived in good health [6]. HALE, like LE, is independent of the size and composition of the population and is therefore useful to make comparisons between populations and over time [5].

A possible application of HALE is to compare cohorts that differ solely in their lifestyle e.g. smoking versus non smoking. Although much work has been done to quantify the current burden of disease attributable to risk factors [1,7-9] HALE estimations conditional on risk factors have not yet been published to our knowledge. Combining LE and HALE estimates conditional on risk factors provides information on whether prevention of obesity and/or smoking would result in compression or expansion of morbidity [2,10], i.e. a decrease or an increase of the period lived with disability. In a previous study Nusselder *et al*. [11] found that eliminating smoking not only extends life but also results in an increase in the number of years lived without disability and thus in compression of morbidity. However, in that study disability was treated as a dichotomous variable (disability or not). Using disability weights attached to disease prevalence rates, different levels of disability caused by different diseases can be aggregated into HALE.

The aims of this study are two-fold:

- present HALE estimates for different cohorts defined conditional on risk factors;

- test whether prevention of obesity and/or smoking results in compression of morbidity.

Using a dynamic population model that calculates the incidence of chronic diseases conditional on epidemiological risk factors, we estimated HALE at age 20 for a cohort of smokers, a cohort of obese people (BMI>30) and a cohort of 'healthy living' people (i.e. non smoking with a BMI < 25). First, it is explained how we estimated HALE using a dynamic population model that combines input from several data sources. Thereafter, results of LE and HALE estimates for the different cohorts are presented. Using the differences between LE and HALE estimates for the different cohorts we will test whether prevention of obesity and smoking results in compression of morbidity. In the last section, implications of our results and methodological issues are discussed.

## Methods

### Basic framework for estimating LE and HALE

To estimate life expectancy (LE) and HALE, the RIVM Chronic Disease Model (CDM) was used [12]. The CDM is a dynamic population model that describes the life course of cohorts in terms of transitions between risk factor classes and changes between disease states over time. Smoking classes distinguished in the CDM are never smokers, current smokers and former smokers. Body weight is modeled in three classes using Body Mass Index (BMI) as indicator: BMI<25 (normal weight), 25 ≤ BMI<30 (overweight), BMI ≥30 (obesity). All model parameters and variables are specified by gender and age. The CDM has been formulated as a set of time-continuous differential equations and the Runge-Kutta method is used to find initial values and numerical approximations for 1 year time steps used in the CDM [13]. The main model outcome variables are incidence, prevalence and mortality numbers, specified by disease, age and gender. The CDM has been used in disease projections and cost effectiveness analyses [14-17].

Using the CDM, we estimated life expectancy (LE) and HALE for three different cohorts:

- a 'healthy living' cohort: a cohort of never smoking men and women aged 20 with a normal weight;

- a 'smoking' cohort: a cohort of men and women aged 20 that smoke throughout their life with a normal weight;

- an 'obese' cohort: a cohort of never smoking men and women aged 20 with a BMI above 30.

The basic formula with which we estimated HALE for the different cohorts is:

HALE=∑tHSV(t)*N(t)N(0)     (1)
 MathType@MTEF@5@5@+=feaafiart1ev1aaatCvAUfKttLearuWrP9MDH5MBPbIqV92AaeXatLxBI9gBaebbnrfifHhDYfgasaacH8akY=wiFfYdH8Gipec8Eeeu0xXdbba9frFj0=OqFfea0dXdd9vqai=hGuQ8kuc9pgc9s8qqaq=dirpe0xb9q8qiLsFr0=vr0=vr0dc8meaabaqaciaacaGaaeqabaqabeGadaaakeaacqWGibascqWGbbqqcqWGmbatcqWGfbqrcqGH9aqpdaWcaaqaamaaqafabaGaemisaGKaem4uamLaemOvayLaeiikaGIaemiDaqNaeiykaKIaeiOkaOIaemOta4KaeiikaGIaemiDaqNaeiykaKcaleaacqWG0baDaeqaniabggHiLdaakeaacqWGobGtcqGGOaakcqaIWaamcqGGPaqkaaGaaCzcaiaaxMaadaqadaqaaiabigdaXaGaayjkaiaawMcaaaaa@48FE@

HALE Health-Adjusted Life Expectancy

*HSV*(*t*) *Health State Valuation of the cohort at time t*

*N*(*t*) *number of survivors of the cohort at time t*

*N*(0) *initial size of the cohort at time 0*

Using the CDM we estimated the number of survivors and the health state valuations corresponding with the time dependent disease status of the different cohorts. For our calculations, we did not take into account transitions between risk factor classes over time. Thus, all cohorts are closed in the sense that no transitions occur between risk factor classes over the life-time.

### Calculating health state valuations

Health state valuations were calculated by coupling disease prevalence rates to disability weights available from the Dutch Burden of Disease study [18]. This specific form of HALE has also been termed disability-adjusted life expectancy (DALE) [19,20]. Disability weights reflect the severity and impact of a disease relative to death and health without diseases and range from 0 (no disability) to 1 (death) [21]. Since the construct of disability encompasses multiple dimensions that are not necessarily of cardinal nature, all valuation methods to scale disability to a 0 to 1 scale imply value choices [21]. The Dutch Burden of Disease Study estimated disability weights of 48 different disease categories, using a large panel of experts and the person trade off method [18] and disease prevalence rates of these different diseases [7].

To estimate comorbidity prevalence, we assumed independence between diseases. Disability weights for comorbidity were defined, assuming a multiplicative model [22], which implies that disability increases with the number of conditions one has, but that the overall effect is less than additive:

HSV(t)=∏d(1−p(d|t)∗w(d))     (2)
 MathType@MTEF@5@5@+=feaafiart1ev1aaatCvAUfKttLearuWrP9MDH5MBPbIqV92AaeXatLxBI9gBaebbnrfifHhDYfgasaacH8akY=wiFfYdH8Gipec8Eeeu0xXdbba9frFj0=OqFfea0dXdd9vqai=hGuQ8kuc9pgc9s8qqaq=dirpe0xb9q8qiLsFr0=vr0=vr0dc8meaabaqaciaacaGaaeqabaqabeGadaaakeaacqWGibascqWGtbWucqWGwbGvcqGGOaakcqWG0baDcqGGPaqkcqGH9aqpdaqeqbqaamaabmaabaGaeGymaeJaeyOeI0IaemiCaaNaeiikaGIaemizaqMaeiiFaWNaemiDaqNaeiykaKIaey4fIOIaem4DaCNaeiikaGIaemizaqMaeiykaKcacaGLOaGaayzkaaaaleaacqWGKbazaeqaniabg+GivdGccaWLjaGaaCzcamaabmaabaGaeGOmaidacaGLOaGaayzkaaaaaa@4BAA@

*p*(*d *| *t*) *prevalence rate of disease d at time t*

*w*(*d*) *disability weight for disease d*

For diseases causally related to BMI and smoking, we used the CDM to estimate time dependent disease prevalence rates (in Appendix A, all diseases related to smoking and/or obesity that are modeled in the CDM are shown). To capture the impact on health state valuations of diseases not causally related to BMI or smoking we used age and gender specific prevalence rates from the Dutch Burden of Disease Study for those diseases and assumed them constant over time.

### Estimating life years and disease prevalence rates with the CDM

For all diseases related to smoking and/or obesity modeled in the CDM, age and sex specific incidence, prevalence and mortality rates were estimated using a three state transition model [23,24]. Formula (3) denotes the change over time in the prevalence rate of disease *d *for a cohort, homogeneous in its risk factor class prevalence, as a function of relative risks, incidence and mortality rates (for notational simplicity, age and sex indices have been omitted in the notation throughout the paper):

dp(d|t)dt
 MathType@MTEF@5@5@+=feaafiart1ev1aaatCvAUfKttLearuWrP9MDH5MBPbIqV92AaeXatLxBI9gBaebbnrfifHhDYfgasaacH8akY=wiFfYdH8Gipec8Eeeu0xXdbba9frFj0=OqFfea0dXdd9vqai=hGuQ8kuc9pgc9s8qqaq=dirpe0xb9q8qiLsFr0=vr0=vr0dc8meaabaqaciaacaGaaeqabaqabeGadaaakeaadaWcaaqaaiabdsgaKjabdchaWjabcIcaOiabdsgaKjabcYha8jabdsha0jabcMcaPaqaaiabdsgaKjabdsha0baaaaa@382C@ = (*i*(*d*)_0 _* *RR*(*d *| *s*_*j*_) * *RR*(*d *| *b*_*k*_) - *em*(*d*) * *p*(*d *| *t*))*(1 - *p*(*d *| *t*))     (3)

*p*(*d *| *t*) *prevalence rate disease d at time t*

*i*(*d*)_0 _*baseline incidence rate disease d for 'healthy living' cohort*

*RR*(*d *| *s*_*j*_) *relative risk for disease d for smoking class j*

*RR*(*d *| *b*_*k*_) *relative risk for disease d for BMI class k*

*em*(*d*) *excess mortality rate disease d*

Risk factors and diseases are linked through relative risks of disease incidence for each risk factor. That is, incidence rates for each risk factor class are found as relative risks times baseline incidence rate. The general assumption used is that conditional on the risk factors included, the disease event rates are independent [25]. For the 'healthy living cohort' relative risks equal one. Appendix B describes how the baseline incidence rate for the 'healthy living' cohort can be derived from incidence rates measured in the general population using relative risks and risk factor class prevalence rates. To estimate incidence, prevalence and mortality rates in the general population, three types of data sources were used: general practitioner registrations, national registries, and population surveys [26,27]. For cancers, national registries were considered the most reliable source. Data for most non-cancer diseases were based on a combination of up to 5 different general practitioner registrations or other medical care registrations. Risk factor prevalence rates for smoking are based on data of STIVORO [28]. For obesity, data from the annual POLS survey from Statistics Netherlands are used [29]. Relative risks on morbidity and mortality for smoking and obesity are based on several observational studies [30-53]. Relative risks of the three BMI classes were calculated in three steps. First, a quadratic function was estimated to describe the non-linear relation between BMI and all cause mortality relative risks for different studies. The parameters of these functions were then plotted against age to estimate an age gradient. In a third step, average relative risks for the three different BMI classes were computed using the BMI distribution within these classes in the Netherlands. For the current and former smoking classes distinguished in the CDM, data were used from studies that reported relative risks for all current and/or all former smokers specified by gender and age. A supplementary file containing input data used in the CDM for our calculations is available online [see Additional file 1].

The CDM describes disease prevalence numbers for each disease separately and it is assumed that the disease-specific attributed mortality rates are additive. Given the relations between disease specific attributed mortality, other causes mortality, disease prevalence rates and relative risks we can describe the change in population numbers needed to estimate life expectancy (see Appendix B for a derivation of the other causes mortality risk):

dN(t)dt=RR(oc|sj)*RR(oc|bk)*m(oc)0*N(t)−∑dam(d)*p(d|t)*N(t)     (4)
 MathType@MTEF@5@5@+=feaafiart1ev1aaatCvAUfKttLearuWrP9MDH5MBPbIqV92AaeXatLxBI9gBaebbnrfifHhDYfgasaacH8akY=wiFfYdH8Gipec8Eeeu0xXdbba9frFj0=OqFfea0dXdd9vqai=hGuQ8kuc9pgc9s8qqaq=dirpe0xb9q8qiLsFr0=vr0=vr0dc8meaabaqaciaacaGaaeqabaqabeGadaaakeaadaWcaaqaaiabdsgaKjabd6eaojabcIcaOiabdsha0jabcMcaPaqaaiabdsgaKjabdsha0baacqGH9aqpcqWGsbGucqWGsbGucqGGOaakcqWGVbWBcqWGJbWycqGG8baFcqWGZbWCdaWgaaWcbaGaemOAaOgabeaakiabcMcaPiabcQcaQiabdkfasjabdkfasjabcIcaOiabd+gaVjabdogaJjabcYha8jabdkgaInaaBaaaleaacqWGRbWAaeqaaOGaeiykaKIaeiOkaOIaemyBa0MaeiikaGIaem4Ba8Maem4yamMaeiykaKYaaSbaaSqaaiabicdaWaqabaGccqGGQaGkcqWGobGtcqGGOaakcqWG0baDcqGGPaqkcqGHsisldaaeqbqaaiabdggaHjabd2gaTjabcIcaOiabdsgaKjabcMcaPiabcQcaQiabdchaWjabcIcaOiabdsgaKjabcYha8jabdsha0jabcMcaPiabcQcaQiabd6eaojabcIcaOiabdsha0jabcMcaPaWcbaGaemizaqgabeqdcqGHris5aOGaaCzcaiaaxMaadaqadaqaaiabisda0aGaayjkaiaawMcaaaaa@758C@

*RR*(*oc *| *s*_*j*_) *relative risk for other causes mortality smoking class j*

*RR*(*oc *| *b*_*k*_) *relative risk for other causes mortality BMI class k*

*m*(*oc*)_0 _*baseline other causes mortality rate for 'healthy living' cohort*

*am*(*d*) *mortality rate attributed to disease d*

The difference of the mortality rates for persons with and without the disease can be interpreted as the excess mortality rate for that disease. However, in a model with multiple diseases these excess mortality rates cannot be interpreted as mortality uniquely attributable to a disease, since the excess mortality rates can also be caused by other co-morbid chronic diseases, e.g. coronary heart disease being a complication of diabetes. Therefore, in the calculation of the prevalence rates excess mortality rates are used, while in the calculation of the number of survivors disease specific attributed mortality rates are used.

### Measuring compression or expansion of morbidity

Differences between LE and HALE indicate the life years that are lost due to ill health and can be interpreted as 'unhealthy' life expectancy or expected years lived with disability. Thus, the ratio HALE/LE can be interpreted as the proportion of life expectancy spent in good health. Absolute compression occurs when 'unhealthy' life expectancy decreases and absolute expansion occurs when 'unhealthy' life expectancy increases. Relative compression occurs when the ratio (LE-HALE)/LE decreases and relative expansion occurs when this ratio increases [6]. Thus, prevention of obesity and smoking results in absolute compression of morbidity if the expected years lived with disability is lowest for the cohort of healthy living people. Relative compression due to prevention occurs if the ratio (LE-HALE)/LE is lowest for healthy living people.

### Sensitivity analyses

In our baseline estimates of LE and HALE it is assumed that relative risks, incidence rates and mortality rates are constant over time. However, in the past, due to medical progress mortality rates of cardiovascular diseases have been declining [54,55]. In a similar fashion, it has been argued that the excess risk of obesity on cardiovascular disease has decreased over time due to better treatment of other risk factors or intermediates like hypertension and diabetes [56,57]. Another crucial factor in HALE calculations and conclusions about compression or expansion of morbidity are the health state valuations of the different cohorts. Since the extra life years gained by successful prevention are lived at high ages the extent to which the health state valuations of the cohorts decrease with age strongly influences conclusions on compression or expansion of morbidity.

To investigate the robustness of our results with respect to future changes in disease epidemiology and the age gradient of the health state valuations we have carried out a series of sensitivity analyses by estimating LE and HALE for the three cohorts in the following scenarios:

- *scenario 1*: a yearly decrease of 1% in attributable mortality rates for all diseases included in the model;

- *scenario 2: *a yearly decrease of 1% in disease incidence rates for all diseases included in the model;

- *scenario 3: *a yearly decrease of 1% in both disease incidence and attributable mortality rates for all diseases included in the model;

- *scenario 4: *a yearly decrease in all relative risks of the obese and smoking cohort using the following formula: *RR*_*t *_= (*RR*_*t*-1_-1)*0.99 + 1 where *RR*_*t *_is the relative risk in year *t*;

- *scenario 5: *health state valuations for all cohorts at all ages were recalculated by subtracting 30% of mean age and sex specific total disability (defined as 1 minus the health state valuation) as estimated using the Dutch Burden of Disease data [22]. This implies a sharper decrease in the health status of the cohorts at older ages. At ages 80 and over this means a reduction larger than 0.1 in the health state valuations of all cohorts.

In scenario 1, 2 and 3 the decrease in mortality and/or incidence rates roughly equals the decrease as used in the Global Burden of Disease projections of global mortality and burden of disease [58]. Scenario 4 reflects the effects of selective disease prevention efforts in smokers and obese as has been observed in the past [56,57]. In scenario 5 we account for the incomplete nature of the Dutch burden of disease: compared to the Global Burden of Disease 2000 study, the diseases selected in the Dutch Burden of Disease study [7] account for only 70% of years lived with disability for European region A [59].

## Results

**Figure 1:**
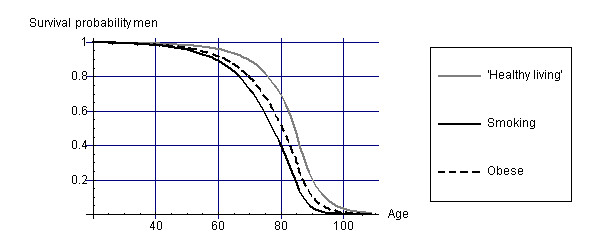
Survival curves for men of the different cohorts.

**Figure 2:**
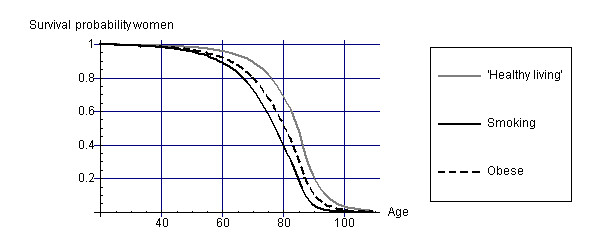
Survival curves for women of the different cohorts.

Figures 1 and 2 display survival curves for the different cohorts for men and women. From the survival curves, it appears that smokers have the lowest life expectancy and that 'healthy living' people have the highest life expectancy.

**Figure 3:**
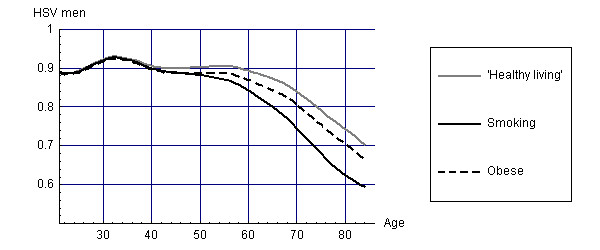
Health State Valuations (HSV) for survivors of different cohorts (men).

**Figure 4:**
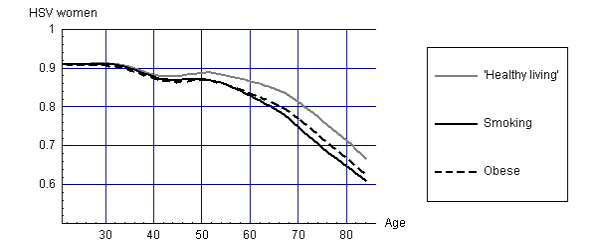
Health State Valuations (HSV) for survivors of different cohorts (women).

For the survivors at different points in time, the disease prevalence rates coupled to disability weights can be used to estimate average health state valuations for the different cohorts. Figure 3 and 4 display these average health state valuations for men and women.

At low ages, health state valuations for all cohorts are similar, but for higher ages health state valuations are lowest for smokers, with differences between smokers and healthy living people increasing up to 0.12. For men, the differences in health state valuations between smokers and obese people are larger than for women. These results suggest that obesity causes relatively more disability in women and smoking more disability in men.

Combining these health state valuations with the life years as displayed in Figures 1 and 2 leads to estimates of HALE as shown in Tables 1 and 2.

**Table 1:** Life Expectancy (LE) and Health Adjusted Life Expectancy (HALE) for men (between brackets: difference with 'healthy living' cohort)

**AGE**	**LE HALE**	**'Healthy living' cohort**	**Smoking cohort**	**Obese cohort**
*20*	*LE*	63.1	55.4	58.5
			(-7.7)	(-4.7)
	*HALE*	54.8	46.9	50.2
			(-7.8)	(-4.6)
*40*	*LE*	44.6	37.1	40.1
			(-7.5)	(-4.5)
	*HALE*	37.0	29.2	32.5
			(-7.8)	(-4.5)
*65*	*LE*	21.0	15.4	17.8
			(-5.6)	(-3.2)
	*HALE*	16.5	9.4	12.5
			(-7.1)	(-4.0)

**Table 2:** Life Expectancy (LE) and Health Adjusted Life Expectancy (HALE) for women (between brackets: difference with 'healthy living' cohort)

**AGE**	**LE HALE**	**'Healthy living' cohort**	**Smoking cohort**	**Obese cohort**
*20*	*LE*	65.7	59.4	61.3
			(-6.3)	(-4.4)
	*HALE*	55.4	49.4	50.8
			(-6.0)	(-4.5)
*40.00*	*LE*	47.0	40.8	42.8
			(-6.2)	(-4.2)
	*HALE*	37.5	31.6	33.1
			(-5.9)	(-4.4)
*65.00*	*LE*	23.2	18.2	20.1
			(-5.0)	(-3.1)
	*HALE*	18.0	12.2	14.0
			(-5.8)	(-4.0)

At age 20 there is a difference in 7.7 years in LE for smoking men compared to healthy men and a difference in LE of 4.7 years for obese men compared to healthy living men. Differences in HALE compared to healthy living men at age 20 are 7.8 years and 4.6 years, for smoking and obese men respectively. At older ages differences in HALE are larger than differences in LE.

Compared to men, decreases in LE and HALE due to smoking and obesity as compared to healthy living are smaller for women. At age 20 there is a difference of 6.3 years in LE for smoking women compared to healthy living women and a difference in LE of 4.4 years for obese women compared to healthy living women. Compared to healthy living women, differences in HALE at age 20 are 6.0 and 4.5 for smoking and obese women, respectively.

Now that LE and HALE have been estimated for the different cohorts, we will address the following question: does prevention of smoking and obesity at young age result in absolute and/or relative compression of morbidity? Table 3 shows that absolute compression occurs due to smoking prevention for men and obesity prevention in women. Absolute expansion occurs for smoking prevention in women and obesity prevention in women. However, successful prevention will always result in relative compression, implying that the period lived with morbidity decreases relative to total life expectancy when prevention policies will be successful.

**Table 3:** Unhealthy life expectancy (LE-HALE) and unhealthy life expectancy relative to life expectancy (LE-HALE)/LE at age 20

		**'Healthy living' cohort**	**Smoking cohort**	**Obese cohort**
*Men*	*LE-HALE*	8.4	8.5	8.3
	*(LE-HALE)/LE*	0.13	0.15	0.14
*Women*	*LE-HALE*	10.3	10.0	10.5
	*(LE-HALE)/LE*	0.16	0.17	0.17

Table 4 displays the results of the sensitivity analysis. Future increases in disease mortality and/or incidence rates increase LE and HALE estimates for all cohorts for both men and women (scenarios 1, 2 and 3). As expected, decreasing relative risks over time results in smaller differences in LE and HALE between the cohorts (scenario 4). Addition of GBD data and a sharper decrease in the health state valuations of all cohorts at older ages lowers HALE estimates substantially (scenario 5). However, differences in HALE between the different cohorts remain substantial in all scenarios: compared to the 'healthy living' cohort HALE estimates of the smoking cohort are minimally 6.3 years lower for men and 4.9 years lower for women. For the obese cohort the minimum differences are 4.0 years and 3.8 years for respectively men and women. In most scenarios 'unhealthy' life expectancies are highest for the 'healthy living' cohort indicating an absolute expansion of morbidity if smoking and/or obesity is prevented. This effect is most pronounced in scenario 5. However, in none of the scenarios does prevention result in relative expansion of morbidity.

**Table 4:** Results of sensitivity analysis: LE, HALE, LE-HALE and (LE-HALE)/LE for men and women at age 20 (between brackets: difference with 'healthy living' cohort)

		**MEN**			**WOMEN**		
		**'Healthy living' cohort**	**Smoking cohort**	**Obese cohort**	**'Healthy living' cohort**	**Smoking cohort**	**Obese cohort**

**Scenario 1**	*LE*	66.4	58.8 (-7.6)	61.2 (-4.2)	68.9	62.1 (-6.7)	64.2 (-4.7)
	*HALE*	57.0	49.2 (-6.8)	52.0 (-5.0)	57.4	51.2 (-6.2)	52.7 (-4.7)
	*LE-HALE*	9.4	9.6	9.2	11.4	10.9	11.5
	*(LE-HALE)/LE*	0.14	0.16	0.15	0.17	0.18	0.18
**Scenario 2**	*LE*	68.0	60.0 (-8.0)	62.5 (-5.5)	69.9	62.9 (-7.0)	65.1 (-4.8)
	*HALE*	59.8	52.3 (-7.5)	54.8 (-5.0)	59.7	53.6 (-6.1)	55.3 (-4.4)
	*LE-HALE*	8.1	7.8	7.7	10.2	9.2	9.8
	*(LE-HALE)/LE*	0.12	0.13	0.12	0.15	0.15	0.15
**Scenario 3**	*LE*	69.9	62.2 (-7.7)	64.2 (-5.7)	71.5	64.3 (-7.2)	66.7 (-4.8)
	*HALE*	61.3	53.9 (-7.4)	56.1 (-5.2)	60.9	54.8 (-6.1)	56.5 (-4.4)
	*LE-HALE*	8.6	8.3	8.0	10.6	9.6	10.3
	*(LE-HALE)/LE*	0.12	0.13	0.12	0.15	0.15	0.15
**Scenario 4**	*LE*	63.1	56.8 (-6.3)	58.9 (-4.2)	65.7	60.3 (-5.4)	61.8 (-3.9)
	*HALE*	54.8	48.5 (-6.3)	50.8 (-4.0)	55.4	50.5 (-4.9)	51.6 (-3.8)
	*LE-HALE*	8.4	8.3	8.1	10.3	9.8	10.3
	*(LE-HALE)/LE*	0.13	0.15	0.14	0.16	0.16	0.17
**Scenario 5**	*LE*	63.1	55.4 (-7.7).	58.5 (-4.7)	65.7	59.4 (-6.4)	61.3 (-4.4)
	*HALE*	51.6	44.5 (-7.1)	47.5 (-4.1)	52.1	46.7 (-5.4)	48.0 (-4.1)
	*LE-HALE*	11.5	10.9	11.0	13.6	12.7	13.3
	*(LE-HALE)/LE*	0.18	0.20	0.19	0.21	0.21	0.22

## Discussion and conclusion

In this paper, estimates of HALE were presented for different cohorts defined conditional on risk factor classes. Estimates of HALE are highest for 'healthy living' people (54.8 for men and 55.4 for women at age 20). Differences in HALE compared to 'healthy living' men at age 20 are 7.8 and 4.6 for respectively smoking and obese men. Differences in HALE compared to 'healthy' women at age 20 are 6.0 and 4.5 for respectively smoking and obese women. At older ages differences in HALE are larger than differences in LE. For all cohorts unhealthy life expectancies are approximately 8 years for men and 10 years for women. As a result, a slight relative compression of morbidity occurs if prevention of smoking and/or obesity is successful. Estimates of LE and HALE and conclusions about compression of morbidity should be made with great caution because of uncertainty with respect to future changes in disease epidemiology. In sensitivity analyses we investigated the sensitivity of our results to future changes in disease epidemiology and to changes in the age gradient of the health state valuations of the different cohorts. Main results of the sensitivity analyses were that although estimates of LE and HALE are sensitive to changes in disease epidemiology, differences in LE and HALE between the different cohorts remain substantial. Furthermore, although a sharper decrease in the health state valuations at older ages results in an absolute expansion of morbidity this does not result in a relative expansion of morbidity. Overall, in most scenarios the proportion of life expectancy spent in good health is a fairly stable proportion for the different cohorts.

It is argued that an incidence based estimate of HALE using a state-transition model is a better indicator than a prevalence based indicator for public health policy since it is not biased by the stock of diseases built up in the past [60]. The drawback of an incidence based methodology is that state-transition models are required for which the data requirements are very large. To minimize data requirements, we only modeled marginal disease prevalence rates, and did not model comorbidity (joint disease prevalence rates). However, as with any modeling study, results depend on the assumptions used in constructing the simulation model and the input data used. We will first discuss the different assumptions and then proceed to discuss the sensitivity of the results in relationship to the input data used.

First of all, we did not distinguish between light and heavy smokers, and BMI was not treated as a continuous variable. The relative risks used for the BMI classes are risk factor class averages calculated using data on the BMI distribution in the Netherlands. Using risk factor class averages in a cohort implies, over age, the most obese tend to die early reducing the mean level of obesity, with age. To what extent the risk factor class averages calculated using the current BMI distributions in the Netherlands can be used as an approximation to simulate average relative risks of a cohort depends on the stability of these distributions over time.

To estimate baseline incidence and mortality rates for the 'healthy living' cohort, we assumed independence between risk factor classes and multiplicative relative risks. Furthermore, relative risks on disease incidence rates are used as an approximation for disease prevalence rates to estimate relative risk for the different cohorts on other causes of death. Although these assumptions may be violated in practice, necessary data to fill this gap are missing.

Another crucial assumption was the one regarding excess mortality risks. Patients with a specific disease have a higher mortality risk than persons without the disease, all other variables equal. The difference in mortality is expressed here as excess mortality which is used in calculations of the disease prevalence rates. However, in general only part of this excess mortality can be attributed to the specific disease, which is used in our calculations of population numbers. The difference between the excess mortality and the part uniquely attributable to the disease can be interpreted as mortality due to co-morbid conditions. The mortality due to co-morbid conditions is especially important on higher ages, such as for COPD for which smoking is an important risk factor with many other related chronic diseases. So part of the COPD excess mortality must be attributed to other smoking related diseases (e.g. coronary heart disease and lung cancer) [61]. Therefore, in the calculation of the prevalence rates excess mortality rates are used, while in the calculation of the number of survivors disease specific attributed mortality rates are used. So far, the problem of excess mortality has received relatively little attention in most population models. Therefore, developing methods to establish relations between excess mortality rates and attributable mortality rates should deserve more attention.

Disability weights for comorbidity were defined assuming a multiplicative adjustment method. We tested for this using alternative weighing methods [22]. Although this affected absolute estimates of HALE it only had a minor influence on differences in HALE. The same also goes for the influence of diseases not causally related to BMI and/or smoking. Excluding them raised HALE estimates, but did not affect substantially the differences found between groups.

Lastly, we assumed that no transitions occur between risk factor classes over time. In reality, of course, transitions between classes do occur: some smokers quit (and some of them might start again later) and obese people of course might lose weight. Moreover, obesity has a more complex age trajectory than smoking in that body composition changes with age [62].

Recently, it was argued that the excess mortality due to obesity had been overestimated and that the effects of obesity attenuate with age, and are not strongly related to mortality above age 70 to 75 [63,64]. Of course, our estimates of LE, and thus also of HALE, would increase for the obese cohort if we imputed relative risks as reported by a study finding lower risks. Other studies, however, reported higher mortality risks associated with obesity [39] which would lead to lower LE estimates for the obese cohort [65]. Moreover, in our analysis, our cohort was defined as being obese but non-smoking. It has been shown that excess mortality due to obesity is highest for never smokers [39,65].

Even though successful prevention would result in health gains this is not necessarily accompanied by a reduction in health care costs. A decline in costs due to risk factor related diseases may well be outweighed by an increase in costs due to risk factor unrelated diseases, especially in life years gained. Prevention, when successful in prolonging life, may therefore cause more costs than it avoids [66,67]. However, this will of course largely depend on the risk factor under study. A next step, therefore, would be to compare the effects of smoking and obesity prevention on health care costs. We conclude that losses in HALE due to smoking and obesity are substantial and that prevention of smoking and obesity can considerably increase both life expectancy and health-adjusted life expectancy. This knowledge underpins the importance of continuing public health policies to prevent unhealthy behavior. However, our results do not indicate that substantial compression of morbidity is to be expected as a result of successful smoking or obesity prevention.

## Competing interests

The author(s) declare that they have no competing interests.

## Authors' contributions

PHMvB carried out the analyses and drafted the initial manuscript. RTH developed the simulation model. All authors contributed to the writing of the paper and approved the final version.

## Appendix A: Diseases related to smoking and obesity in the CDM (see Table 5)

**Table 5:** diseases modeled in the CDM and their relation to smoking and obesity

	**Related to smoking**	**Related to obesity**
**Cardiovascular disease**		
*Acute myocardial infarct (AMI)*	+	+
*Angina pectoris*	+	+
*Chronic Heart Failure*	+	+
*Stroke (CVA)*	+	+
**Cancer**		
*Lung*	+	
*Stomach*	+	
*Oesophagus*	+	
*Pancreas*	+	
*Oral cavity*	+	
*Larynx*	+	
*Uriny bladder*	+	
*Kidney*	+	+
*Rectum*		+
*Colon*		+
*Breast*		+
*Prostate*		+
*Endometrium*		+
**Other**		
*COPD*	+	
*Diabetes*	+	+
*Atrhrosis of the hip*		+
*Arthrosis of the knee*		+
*Dorsopathies (low back pain)*		+

## Appendix B: Calculating baseline mortality and incidence rates with the CDM

### Calculating baseline mortality rates and risk factor class specific mortality rates

Mortality rates from Statistics Netherlands for the year 2004 [29] are attributed to risk factor classes to derive mortality rates specified by risk factor class. Assuming independence between risk factor class prevalence rates and multiplicative relative risks (i.e. no interaction on log-linear scale) we can write mortality rates for the different cohorts as:

*m*(*tot *| *s*_*i*_, *b*_*j*_) = *m*(*tot*)_0 _* *RR*(*tot *| *s*_*j*_) * *RR*(*tot *| *b*_*k*_)     (B1.1)

*m*(*tot *| *s*_*i*_, *b*_*j*_) *all cause mortality rate for cohort for smoking class j BMI class k*

*m*(*tot*)_0 _*baseline all cause mortality rate for 'healthy living' cohort*

*RR*(*tot *| *s*_*j*_) *relative risk all cause mortality smoking class j*

*RR*(*tot *| *b*_*k*_) *relative risk all cause mortality BMI class k*

Using (B1.1) we can write the baseline mortality rate for the 'healthy living' cohort as:

m(tot)0=m(tot)∑j,kRR(tot|sj)*RR(tot|bk)*sj*bk     (B1.2)
 MathType@MTEF@5@5@+=feaafiart1ev1aaatCvAUfKttLearuWrP9MDH5MBPbIqV92AaeXatLxBI9gBaebbnrfifHhDYfgasaacH8akY=wiFfYdH8Gipec8Eeeu0xXdbba9frFj0=OqFfea0dXdd9vqai=hGuQ8kuc9pgc9s8qqaq=dirpe0xb9q8qiLsFr0=vr0=vr0dc8meaabaqaciaacaGaaeqabaqabeGadaaakeaacqWGTbqBcqGGOaakcqWG0baDcqWGVbWBcqWG0baDcqGGPaqkdaWgaaWcbaGaeGimaadabeaakiabg2da9maalaaabaGaemyBa0MaeiikaGIaemiDaqNaem4Ba8MaemiDaqNaeiykaKcabaWaaabuaeaacqWGsbGucqWGsbGucqGGOaakcqWG0baDcqWGVbWBcqWG0baDcqGG8baFcqWGZbWCdaWgaaWcbaGaemOAaOgabeaakiabcMcaPiabcQcaQiabdkfasjabdkfasjabcIcaOiabdsha0jabd+gaVjabdsha0jabcYha8jabdkgaInaaBaaaleaacqWGRbWAaeqaaOGaeiykaKcaleaacqWGQbGAcqGGSaalcqWGRbWAaeqaniabggHiLdGccqGGQaGkcqWGZbWCdaWgaaWcbaGaemOAaOgabeaakiabcQcaQiabdkgaInaaBaaaleaacqWGRbWAaeqaaaaakiaaxMaacaWLjaWaaeWaaeaacqqGcbGqcqaIXaqmcqGGUaGlcqaIYaGmaiaawIcacaGLPaaaaaa@6C0F@

*m*(*tot*) *all cause mortality rate (Statistics Netherlands)*

*s*_*j *_*prevalence rate smoking class j*

*b*_*j *_*prevalence rate BMI class k*

### Calculating baseline disease incidence rates and risk factor class specific disease incidence rates

*i*(*d *| *s*_*j*_, *b*_*k*_) = *i*(*d*)_0 _* *RR*(*d *| *s*_*j*_) * *RR*(*d *| *b*_*k*_)     (B2.1)

*i*(*d *| *s*_*i*_, *b*_*j*_) *incidence rate disease d for cohort smoking class j BMI class k*

*i*(*d*)_0 _*baseline incidence rate for 'healthy' cohort*

*RR*(*d *| *s*_*j*_) *relative risk for disease d smoking class j*

*RR*(*d *| *b*_*k*_) *relative risk for disease d BMI class k*

i(d)0=i(d)∑j,kRR(d|sj)*RR(d|bk)*sj*bk     (B2.2)
 MathType@MTEF@5@5@+=feaafiart1ev1aaatCvAUfKttLearuWrP9MDH5MBPbIqV92AaeXatLxBI9gBaebbnrfifHhDYfgasaacH8akY=wiFfYdH8Gipec8Eeeu0xXdbba9frFj0=OqFfea0dXdd9vqai=hGuQ8kuc9pgc9s8qqaq=dirpe0xb9q8qiLsFr0=vr0=vr0dc8meaabaqaciaacaGaaeqabaqabeGadaaakeaacqWGPbqAcqGGOaakcqWGKbazcqGGPaqkdaWgaaWcbaGaeGimaadabeaakiabg2da9maalaaabaGaemyAaKMaeiikaGIaemizaqMaeiykaKcabaWaaabuaeaacqWGsbGucqWGsbGucqGGOaakcqWGKbazcqGG8baFcqWGZbWCdaWgaaWcbaGaemOAaOgabeaakiabcMcaPiabcQcaQiabdkfasjabdkfasjabcIcaOiabdsgaKjabcYha8jabdkgaInaaBaaaleaacqWGRbWAaeqaaOGaeiykaKIaeiOkaOIaem4Cam3aaSbaaSqaaiabdQgaQbqabaGccqGGQaGkcqWGIbGydaWgaaWcbaGaem4AaSgabeaaaeaacqWGQbGAcqGGSaalcqWGRbWAaeqaniabggHiLdaaaOGaaCzcaiaaxMaadaqadaqaaiabbkeacjabikdaYiabc6caUiabikdaYaGaayjkaiaawMcaaaaa@600C@

*i*(*d*) *population incidence rate disease d*

### Calculating baseline risk factor class specific relative risk for other causes mortality

The CDM describes disease prevalence numbers for each disease separately and it is assumed that the disease-specific attributed mortality rates are additive. The all cause mortality rates are the sum of the disease specific attributed mortality rates and the mortality rates from other causes of death:

m(oc)=m(tot)−∑dam(d)p(d)     (B3.1)
 MathType@MTEF@5@5@+=feaafiart1ev1aaatCvAUfKttLearuWrP9MDH5MBPbIqV92AaeXatLxBI9gBaebbnrfifHhDYfgasaacH8akY=wiFfYdH8Gipec8Eeeu0xXdbba9frFj0=OqFfea0dXdd9vqai=hGuQ8kuc9pgc9s8qqaq=dirpe0xb9q8qiLsFr0=vr0=vr0dc8meaabaqaciaacaGaaeqabaqabeGadaaakeaacqWGTbqBcqGGOaakcqWGVbWBcqWGJbWycqGGPaqkcqGH9aqpcqWGTbqBcqGGOaakcqWG0baDcqWGVbWBcqWG0baDcqGGPaqkcqGHsisldaaeqbqaaiabdggaHjabd2gaTjabcIcaOiabdsgaKjabcMcaPiabdchaWjabcIcaOiabdsgaKjabcMcaPaWcbaGaemizaqgabeqdcqGHris5aOGaaCzcaiaaxMaadaqadaqaaiabbkeacjabiodaZiabc6caUiabigdaXaGaayjkaiaawMcaaaaa@5003@

*m*(*oc*) *mortality rate for other causes of death*

*p*(*d*) *disease d prevalence rates (several sources)*

*am*(*d*) *mortality rate attributed to disease d*

Mortality rates attributed to diseases are calculated by dividing the cause specific mortality rates registered by Statistics Netherlands by disease specific prevalence rates:

am(d)=c(d)p(d)     (B3.2)
 MathType@MTEF@5@5@+=feaafiart1ev1aaatCvAUfKttLearuWrP9MDH5MBPbIqV92AaeXatLxBI9gBaebbnrfifHhDYfgasaacH8akY=wiFfYdH8Gipec8Eeeu0xXdbba9frFj0=OqFfea0dXdd9vqai=hGuQ8kuc9pgc9s8qqaq=dirpe0xb9q8qiLsFr0=vr0=vr0dc8meaabaqaciaacaGaaeqabaqabeGadaaakeaacqWGHbqycqWGTbqBcqGGOaakcqWGKbazcqGGPaqkcqGH9aqpdaWcaaqaaiabdogaJjabcIcaOiabdsgaKjabcMcaPaqaaiabdchaWjabcIcaOiabdsgaKjabcMcaPaaacaWLjaGaaCzcamaabmaabaGaeeOqaiKaeG4mamJaeiOla4IaeGOmaidacaGLOaGaayzkaaaaaa@42D3@

*c*(*d*) *cause specific mortality rate disease d*

It is assumed that for any disease the attributed mortality is independent from the risk factor levels. This means that the risk factors affect the disease prognosis only through increased risks for other diseases and mortality from other causes of death. Using the relative risk for the incidence of diseases as an approximation for relative risk for the prevalence of diseases we calculated the relative risks for other causes of death:

RR(oc|sj)=RR(tot|sj)*m(tot)0S−∑dRR(d|sj)*am(d)*p(d)0Sm(oc)0S     (B3.3)
 MathType@MTEF@5@5@+=feaafiart1ev1aaatCvAUfKttLearuWrP9MDH5MBPbIqV92AaeXatLxBI9gBaebbnrfifHhDYfgasaacH8akY=wiFfYdH8Gipec8Eeeu0xXdbba9frFj0=OqFfea0dXdd9vqai=hGuQ8kuc9pgc9s8qqaq=dirpe0xb9q8qiLsFr0=vr0=vr0dc8meaabaqaciaacaGaaeqabaqabeGadaaakeaacqWGsbGucqWGsbGucqGGOaakcqWGVbWBcqWGJbWycqGG8baFcqWGZbWCdaWgaaWcbaGaemOAaOgabeaakiabcMcaPiabg2da9maalaaabaGaemOuaiLaemOuaiLaeiikaGIaemiDaqNaem4Ba8MaemiDaqNaeiiFaWNaem4Cam3aaSbaaSqaaiabdQgaQbqabaGccqGGPaqkcqGGQaGkcqWGTbqBcqGGOaakcqWG0baDcqWGVbWBcqWG0baDcqGGPaqkdaWgaaWcbaGaeGimaaJaem4uamfabeaakiabgkHiTmaaqafabaGaemOuaiLaemOuaiLaeiikaGIaemizaqMaeiiFaWNaem4Cam3aaSbaaSqaaiabdQgaQbqabaGccqGGPaqkcqGGQaGkcqWGHbqycqWGTbqBcqGGOaakcqWGKbazcqGGPaqkcqGGQaGkcqWGWbaCcqGGOaakcqWGKbazcqGGPaqkdaWgaaWcbaGaeGimaaJaem4uamfabeaaaeaacqWGKbazaeqaniabggHiLdaakeaacqWGTbqBcqGGOaakcqWGVbWBcqWGJbWycqGGPaqkdaWgaaWcbaGaeGimaaJaem4uamfabeaaaaGccaWLjaGaaCzcamaabmaabaGaeeOqaiKaeG4mamJaeiOla4IaeG4mamdacaGLOaGaayzkaaaaaa@7B89@

p(d)0S=p(d)∑jRR(d|sj)*sj     (B3.4)
 MathType@MTEF@5@5@+=feaafiart1ev1aaatCvAUfKttLearuWrP9MDH5MBPbIqV92AaeXatLxBI9gBaebbnrfifHhDYfgasaacH8akY=wiFfYdH8Gipec8Eeeu0xXdbba9frFj0=OqFfea0dXdd9vqai=hGuQ8kuc9pgc9s8qqaq=dirpe0xb9q8qiLsFr0=vr0=vr0dc8meaabaqaciaacaGaaeqabaqabeGadaaakeaacqWGWbaCcqGGOaakcqWGKbazcqGGPaqkdaWgaaWcbaGaeGimaaJaem4uamfabeaakiabg2da9maalaaabaGaemiCaaNaeiikaGIaemizaqMaeiykaKcabaWaaabuaeaacqWGsbGucqWGsbGucqGGOaakcqWGKbazcqGG8baFcqWGZbWCdaWgaaWcbaGaemOAaOgabeaakiabcMcaPiabcQcaQiabdohaZnaaBaaaleaacqWGQbGAaeqaaaqaaiabdQgaQbqab0GaeyyeIuoaaaGccaWLjaGaaCzcamaabmaabaGaeeOqaiKaeG4mamJaeiOla4IaeGinaqdacaGLOaGaayzkaaaaaa@50C5@

m(oc)0S=m(oc)∑jRR(oc|sj)*sj     (B3.5)
 MathType@MTEF@5@5@+=feaafiart1ev1aaatCvAUfKttLearuWrP9MDH5MBPbIqV92AaeXatLxBI9gBaebbnrfifHhDYfgasaacH8akY=wiFfYdH8Gipec8Eeeu0xXdbba9frFj0=OqFfea0dXdd9vqai=hGuQ8kuc9pgc9s8qqaq=dirpe0xb9q8qiLsFr0=vr0=vr0dc8meaabaqaciaacaGaaeqabaqabeGadaaakeaacqWGTbqBcqGGOaakcqWGVbWBcqWGJbWycqGGPaqkdaWgaaWcbaGaeGimaaJaem4uamfabeaakiabg2da9maalaaabaGaemyBa0MaeiikaGIaem4Ba8Maem4yamMaeiykaKcabaWaaabuaeaacqWGsbGucqWGsbGucqGGOaakcqWGVbWBcqWGJbWycqGG8baFcqWGZbWCdaWgaaWcbaGaemOAaOgabeaakiabcMcaPiabcQcaQiabdohaZnaaBaaaleaacqWGQbGAaeqaaaqaaiabdQgaQbqab0GaeyyeIuoaaaGccaWLjaGaaCzcamaabmaabaGaeeOqaiKaeG4mamJaeiOla4IaeGynaudacaGLOaGaayzkaaaaaa@54EA@

m(tot)0S=m(tot)∑jRR(tot|sj)*sj
 MathType@MTEF@5@5@+=feaafiart1ev1aaatCvAUfKttLearuWrP9MDH5MBPbIqV92AaeXatLxBI9gBaebbnrfifHhDYfgasaacH8akY=wiFfYdH8Gipec8Eeeu0xXdbba9frFj0=OqFfea0dXdd9vqai=hGuQ8kuc9pgc9s8qqaq=dirpe0xb9q8qiLsFr0=vr0=vr0dc8meaabaqaciaacaGaaeqabaqabeGadaaakeaacqWGTbqBcqGGOaakcqWG0baDcqWGVbWBcqWG0baDcqGGPaqkdaWgaaWcbaGaeGimaaJaem4uamfabeaakiabg2da9maalaaabaGaemyBa0MaeiikaGIaemiDaqNaem4Ba8MaemiDaqNaeiykaKcabaWaaabuaeaacqWGsbGucqWGsbGucqGGOaakcqWG0baDcqWGVbWBcqWG0baDcqGG8baFcqWGZbWCdaWgaaWcbaGaemOAaOgabeaakiabcMcaPiabcQcaQiabdohaZnaaBaaaleaacqWGQbGAaeqaaaqaaiabdQgaQbqab0GaeyyeIuoaaaaaaa@52F1@

*RR*(*oc *| *s*_*j*_) *relative risk for other cause mortality smoking class j*

*m*(*oc*)_0*s *_*baseline other cause mortality rate for non smoking cohort*

*p*(*d*)_0*s *_*baseline prevalence rate disease d for non smoking cohort*

*m*(*tot*)_0*s *_*baseline all cause mortality rate for non smoking cohort*

These equations can be solved for *RR*(*oc *| *s*_*j*_,) by substituting equations (B3.4) and (B3.5) into equation (B3.3). In a similar fashion, relative risks for other causes mortality of for overweight and obesity can be derived. Given *RR*(*oc *| *s*_*j*_,) and *RR*(*oc *| *b*_*k*_,) the baseline other cause mortality rate can be found:

*RR*(*oc *| *s*_*j*_, *b*_*k*_) = *RR*(*oc *| *s*_*j*_) * *RR*(*oc *| *b*_*k*_)     (B3.6)

m(oc)0=m(oc)∑j,kRR(oc|sj)*RR(oc|bk)*sj*bk     (B3.7)
 MathType@MTEF@5@5@+=feaafiart1ev1aaatCvAUfKttLearuWrP9MDH5MBPbIqV92AaeXatLxBI9gBaebbnrfifHhDYfgasaacH8akY=wiFfYdH8Gipec8Eeeu0xXdbba9frFj0=OqFfea0dXdd9vqai=hGuQ8kuc9pgc9s8qqaq=dirpe0xb9q8qiLsFr0=vr0=vr0dc8meaabaqaciaacaGaaeqabaqabeGadaaakeaacqWGTbqBcqGGOaakcqWGVbWBcqWGJbWycqGGPaqkdaWgaaWcbaGaeGimaadabeaakiabg2da9maalaaabaGaemyBa0MaeiikaGIaem4Ba8Maem4yamMaeiykaKcabaWaaabuaeaacqWGsbGucqWGsbGucqGGOaakcqWGVbWBcqWGJbWycqGG8baFcqWGZbWCdaWgaaWcbaGaemOAaOgabeaakiabcMcaPiabcQcaQiabdkfasjabdkfasjabcIcaOiabd+gaVjabdogaJjabcYha8jabdkgaInaaBaaaleaacqWGRbWAaeqaaOGaeiykaKIaeiOkaOIaem4Cam3aaSbaaSqaaiabdQgaQbqabaGccqGGQaGkcqWGIbGydaWgaaWcbaGaem4AaSgabeaaaeaacqWGQbGAcqGGSaalcqWGRbWAaeqaniabggHiLdaaaOGaaCzcaiaaxMaadaqadaqaaiabbkeacjabiodaZiabc6caUiabiEda3aGaayjkaiaawMcaaaaa@65BC@

*RR*(*oc *| *s*_*j*_, *b*_*k*_) *relative risk for other cause mortality smoking class j BMI class k*

*RR*(*oc *| *b*_*k*_) *relative risk for other cause mortality BMI class k*

*m*(*oc*)_0 _*baseline other cause mortality rate for 'healthy living' cohort*

*p*(*d*)_0 _*baseline prevalence rate disease d for 'healthy living' cohort*

## Supplementary Material

Additional file 1 Excel file containing input data of the RIVM Chronic Disease Model Click here for file
